# Determining Light Intensity, Timing and Type of Visible and Circadian Light From an Ambulatory Circadian Monitoring Device

**DOI:** 10.3389/fphys.2019.00822

**Published:** 2019-06-26

**Authors:** Raquel Arguelles-Prieto, Maria-Angeles Bonmati-Carrion, Maria Angeles Rol, Juan Antonio Madrid

**Affiliations:** ^1^Chronobiology Lab, Department of Physiology, College of Biology, University of Murcia, Mare Nostrum Campus, IUIE, IMIB-Arrixaca, Murcia, Spain; ^2^Centro de Investigación Biomédica en Red Fragilidad y Envejecimiento Saludable (CIBERFES), Madrid, Spain

**Keywords:** circadian light, ambulatory circadian monitoring, chronobiology, Kronowise^®^, melanopsin, ipRGCs

## Abstract

During last decades, the way of life in modern societies has deeply modified the temporal adjustment of the circadian system, mainly due to the inappropriate use of artificial lighting and the high prevalence of social jet-lag. Therefore, it becomes necessary to design non-invasive and practical tools to monitor circadian marker rhythms but also its main synchronizer, the light-dark cycle under free-living conditions. The aim of this work was to improve the ambulatory circadian monitoring device (ACM, Kronowise^®^) capabilities by developing an algorithm that allows to determine light intensity, timing and circadian light stimulation by differentiating between full visible, infrared and circadian light, as well as to discriminate between different light sources (natural and artificial with low and high infrared composition) in subjects under free living conditions. The ACM device is provided with three light sensors: (i) a wide-spectrum sensor (380–1100 nm); (ii) an infrared sensor (700–1100 nm) and (iii) a sensor equipped with a blue filter that mimics the sensitivity curve of the melanopsin photopigment and the melatonin light suppression curve. To calibrate the ACM device, different commercial light sources and sunlight were measured at four different standardized distances with both a spectroradiometer (SPR) and the ACM device. CIE S 026/E:2018 (2018), toolbox software was used to calculate the melanopic stimulation from data recorded by SPR. Although correlation between raw data of luminance measured by ACM and SPR was strong for both full spectrum (*r* = 0.946, *p* < 0.0001) and circadian channel (*r* = 0.902, *p* < 0.0001), even stronger correlations were obtained when light sources were clustered in three groups: natural, infrared-rich artificial light and infrared-poor artificial light, and their corresponding linear correlations with SPR were considered (*r* = 0.997, *p* < 0.0001 and *r* = 0.998, *p* < 0.0001, respectively). Our results show that the ACM device provided with three light sensors and the algorithm developed here allow an accurate detection of light type, intensity and timing for full visible and circadian light, with simultaneous monitoring of several circadian marker rhythms that will open the possibility to explore light synchronization in population groups while they maintain their normal lifestyle.

## Introduction

Since the appearance of life on Earth, endogenous cellular mechanisms to keep track of time allowed individuals to anticipate and adapt to cyclical environmental changes, assuring that biochemical, physiological and behavioral processes occur daily at a species-specific appropriate time, including timing of sleep and wake. This internal synchronization to environmental cues is a fundamental requirement for the survival of living beings (Panda et al., 2002). In mammals, the circadian system consists of a hierarchically organized network of structures driven by a circadian pacemaker located at the suprachiasmatic nuclei of the hypothalamus, which is responsible of sending temporary rhythmic signals to a variety of organs and tissues (Garaulet and Madrid, 2010). Under natural conditions, these circadian rhythms are entrained to a 24-h cycle by “zeitgebers” (literally, “time givers”), among which the most powerful is the light-dark cycle (Roenneberg and Foster, 1997). This “zeitgeber” synchronizes the circadian system through the intrinsically photosensitive retinal ganglion cells (ipRGCs), which contain melanopsin, a photopigment sensitive to 460–480 nm light (Brainard et al., 2001; Berson et al., 2002).

With the invention of electrical lighting, about 150 years ago, light exposure patterns have been modified and, therefore, temporal adjustment of the circadian system has been deeply altered (Reiter et al., 2012; Bonmati-Carrion et al., 2014a). Technological advances in lighting as well as lifestyle in modern societies, involving light at night, excessive indoor time, shift work, leisure activities during nighttime, traveling around the world, and specially the use of inappropriate artificial lighting, have completely altered our physiology, giving rise to a wide variety of health concerns of circadian origin (Pauley, 2004; Navara and Nelson, 2007; Erren and Reiter, 2009; Ortiz-Tudela et al., 2012). They are included in the global concept “chronodisruption” (CD), defined as the temporary or chronic state of internal desynchronization, either between different circadian rhythms or between exogenous and endogenous circadian components (Erren and Reiter, 2009).

To evaluate the impact of lighting conditions on circadian system it seems necessary to develop new, non-invasive and practical tools to detect light-dark chronodisruptive patterns and allow the treatment of patients with circadian disorders. In the last few years, many studies have focused on ambulatory monitoring of circadian rhythms in humans. However, only some of them reported visible light exposure (Berger et al., 2010; Kolodyazhniy et al., 2011; Bonmati-Carrion et al., 2014b; Lemmer et al., 2016), and very few included melanopic stimulation (Bierman et al., 2005; Figueiro et al., 2013; Cao et al., 2015). Thus, our laboratory proposed a few years ago, for the first time, the integration of three variables (skin temperature, activity and position) together with light exposure in one device, in order to evaluate globally, that is considering both input and output signals, the status of the human circadian system under normal living conditions (Ortiz-Tudela et al., 2010; Bonmati-Carrion et al., 2014b).

To advance in implementing these ambulatory techniques, we developed an ACM device that includes both visible and blue light exposure monitoring, together with other circadian marker rhythms (time in movement, motor acceleration, distal skin temperature, and body position) to improve the assessment of sleep-wake rhythms and circadian system functionality, named Kronowise^®^ (Kronohealth SL, University of Murcia, Spain) (Madrid-Navarro et al., 2018).

Therefore, the objective of this work was to improve and validate new ACM features to allow the non-invasive recording of full visible and circadian light under free-living conditions. To this, firstly, we selected a blue filter that mimics the melanopic spectrum; secondly, SPR and ACM light recordings were compared in order to calibrate ACM sensors and thirdly, algorithms to differentiate between natural and artificial light exposure as well as to calculate the precise full visible and circadian photostimulation were developed.

## Materials and Methods

### Ambulatory Circadian Monitoring Device

**FIGURE 1:**
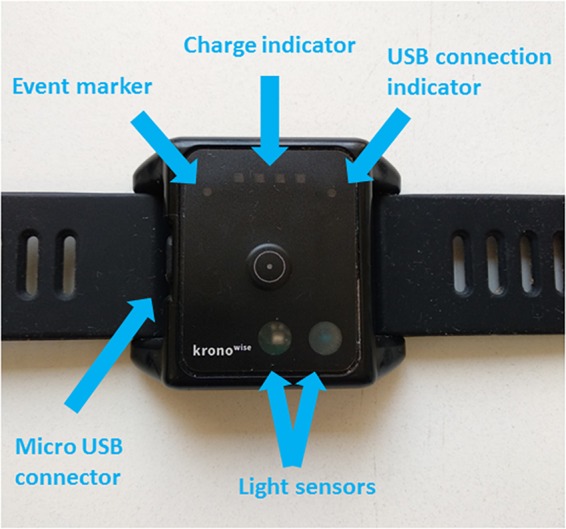
Kronowise^®^ ACM device.

**FIGURE 2:**
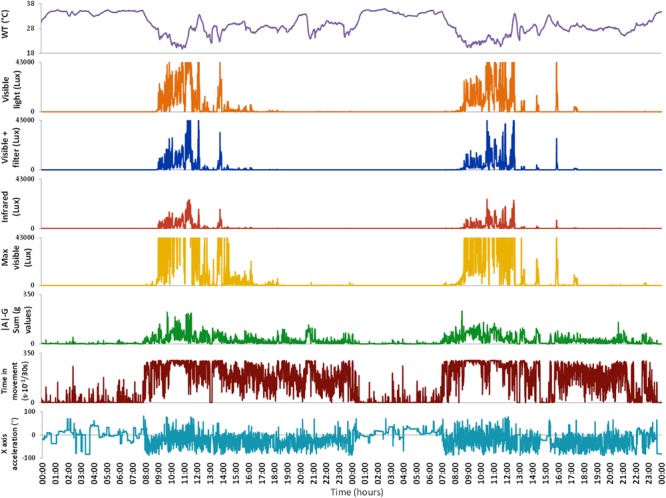
Variables monitored by Kronowise^®^ as exported by its programming software (Kronoware 10.0) from a representative recording. From top to bottom: skin wrist temperature (in °C), visible light, blue filtered light, infrared light, maximum visible light (all in lux), integrated acceleration (expressed in *g-*values), integrated time in movement (expressed as motion events in 30 s epochs) and tilt of *X* axis in grades. Time in movement graph is clipped because, under natural conditions, it could never exceed 300 s × 10^-1^/30 s. Light variables are clipped because the device is linear up to 43000 lux and above that it gets saturated.

**FIGURE 3:**
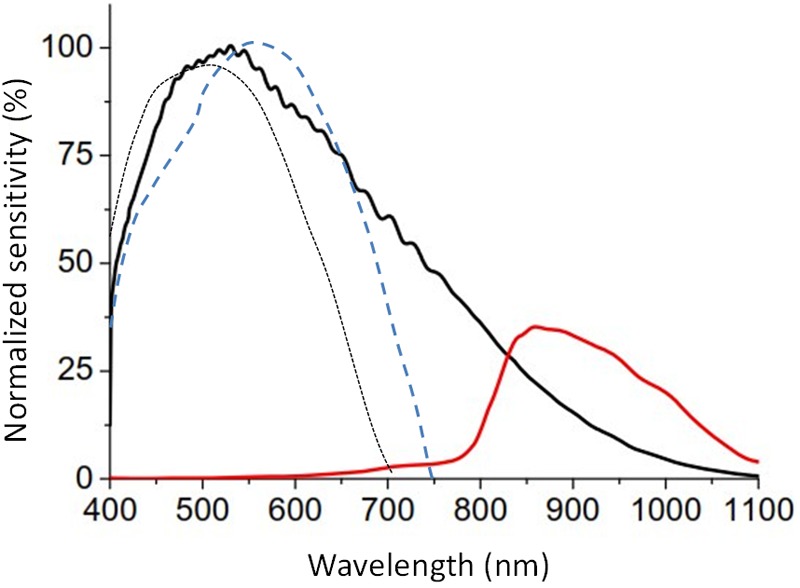
Relative spectral response of full (black line) and infrared channels (red line) of the visible light sensor of ACM Kronowise provided by the manufacturer, compared with the normalized curve of human photopic (blue dashed line, Vos, 1978) and scotopic vision (black dotted line, CIE, 1951).

To perform this study we used an ambulatory circadian monitoring (ACM) device developed by our group: Kronowise^®^ (Kronohealth SL, Spain) (Figure 1). Kronowise^®^ is a wrist device that provides information about different variables related to circadian rhythms, as shown in Figure 2. It weighs approximately 60 g, its battery lasts up to 21 days, and it includes (Madrid-Navarro et al., 2018):

– A temperature sensor, with a precision of ±0.1°C at 25°C and a resolution of 0.0635°C.
– A triaxial calibrated MEMS-accelerometer with a linear and equal sensitivity along the three axes, with a range of ±2 g and a sensitivity of 0.001 g. The default sampling frequency was set at 10 Hz.
– Three light sensors, on the front, determine full spectrum, infrared, and blue light, with a range of between 0.01 and 43,000 lux, 16 bits of resolution, an internal auto-setting according to the luminance level, and suppression of flicker at 50/60 Hz. The infrared sensor was sensitive to radiation from 800 to 1,070 nm, whereas the blue light detector was equipped with a Gaussian filter, which eliminates all visible radiation below 440 and over 500 nm (Figure 3) but let pass the infrared radiation. Furthermore, integration time for measurements can be configured at 50, 100, and 400 ms, although when not specified it is predetermined at 100 ms (enough to filter flicker noise, typical of fluorescent lamps).

Communication between a computer and Kronowise^®^ was established using Kronoware 10.0 software (Kronohealth SL, Spain) *via* a USB port to allow data extraction. This software allows visual inspection of the data before its analysis to eliminate possible artifacts, and the calculation of basic circadian and sleep parameters. The coefficient of variation intra-device is 0.11% and inter-devices is 2.5% for visible light, while for blue light are 0.14% and 3.14%, respectively.

### Photometric Performance of Light Channels

Kronowise^®^ light sensors sensitivity in the visible light spectrum is quite similar to the photopic sensitivity of human retina and its sensitivity remains stable throughout all human photopic light channels (blue, green or red, Figure 3).

**FIGURE 4:**
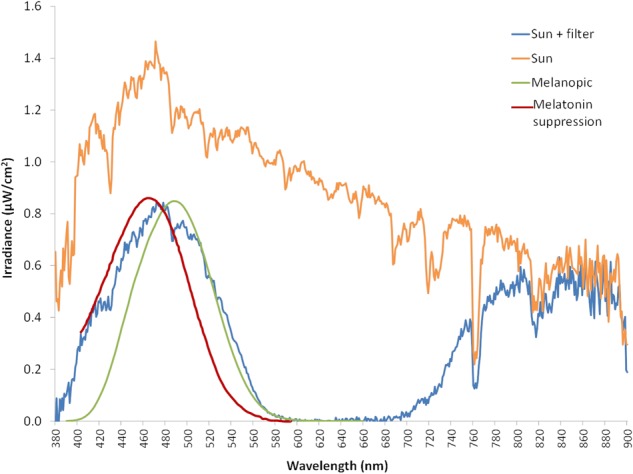
Sun light spectrum measured in irradiance with a spectroradiometer (orange line). Blue line represents the sun light spectrum filtered by the blue filter (also measured with the spectroradiometer), while green line corresponds to ipRGCs sensitivity curve according to the model from Enezi et al. (2011), and red line indicates melatonin suppression curve by light according to the model from Brainard et al. (2001).

**FIGURE 5:**
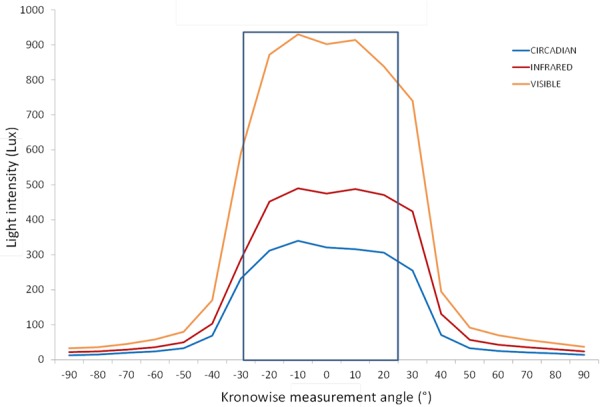
Light intensity according to the measurement angle for the three light Kronowise channels from a light source placed at 50 cm from the device. More than an 80% of maximal light intensity was detected by the three channels in a range of 50°. Blue line represents the circadian channel recordings, the red line denotes the infrared channel recordings, and the orange line corresponds to the visible channel recordings.

Blue filter was selected among several ones to mimic melanopic and melatonin inhibitory response by light. Figure 4 shows the spectral irradiance of sunlight when passing through the filter as measured by the SPR. The light sensors’ directional response in one plane is close to a Gaussian curve with no selective shadowing by incident angle, since more than 80% of maximal light intensity is detected in the range of central 50°, and as measurement angle increases in both directions, sensitivity drops drastically (Figure 5), which resembles the three-dimensional sensitivity curve of the human eye to incident light (Figueiro et al., 2013). This is because light sensors are located in the center of a window covered by an opal glass diffuser.

### Lighting Assessment

ACM light sensors were calibrated by a visible-near infrared spectroradiometer (SPR) (Ocean Optics Inc., FL, United States) used as reference device. This SPR measures several parameters as those used in this study: irradiance and illuminance. Besides, it incorporates a cosine corrector (model CC-3, Ocean Optics Inc., FL, United States) with an opaline glass as diffuser and measurement angle of 180°.

**FIGURE 6:**
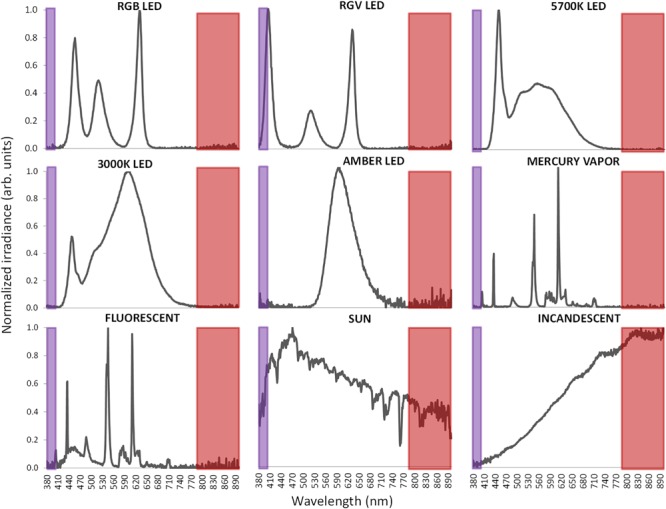
Normalized visible light spectra from the nine different light sources used in this study measured with spectroradiometer. Normalization has been performed to the maximum peak of irradiance. Infrared band is highlighted as a red area and ultraviolet band in violet.

Light exposure under laboratory conditions was performed with a variety of commercial artificial lighting sources including (Figure 6): 5700 K LED (LuciPanel Evo from Lucibel SA, France), 3000 K LED (LuciPanel Evo from Lucibel SA, France), amber LED (Ignialight Sacopa S.A.U., Spain), red-green-blue (RGB) LED (prototype made in University of Murcia), red-green-violet (RGV) LED (prototype made in University of Murcia, Spain), mercury vapor (Luxten, Romania), incandescent (KDE Group, Spain), fluorescent (ADEO, France) and sunlight measured from a south-west facing window between 11 AM and 12:20 PM during a partly cloudy day.

For measurements, room was completely in darkness with the exception of the light to be assessed. Four different distances from the light source to SPR and ACM were selected: 54, 180, 237, and 480 cm.

### Development of the Algorithm for Light Exposure Monitoring

Since ACM light sensors are sensitive to infrared radiation (Figure 3) and measured light sources present very different spectra, including in some cases a substantial part in the infrared band of the spectrum, it becomes necessary to consider infrared radiation in order to adjust measurements to visible and circadian light.

The algorithm for the calculation of visible and circadian light exposure and discriminating light source was developed according to the following process:

(i) For each light source and distance, lighting measurements were recorded by SPR and ACM simultaneously. Each point was measured three times and expressed as the average for both devices.
(ii) Correlations between SPR and ACM visible light measurements for each light source were calculated.
(iii) Then, the proportion of infrared *versus* full light spectrum (IR/Full ratio) was calculated from data recorded by ACM full and infrared sensors.
(iv) Three groups of lights sources were obtained based on IR/Full ratio allowing us to discriminate the following light categories: artificial lights with low IR content, artificial lights with high IR content and sun light.
(v) For each one of these three categories, correlations between SPR and ACM visible light measurements were performed again.
(vi) Thus, for any given lighting device, lights are firstly classified according to its IR/Full ratio into one of the three afore mentioned categories and then, the adequate correlation equation for light calculation was applied.

The same process was performed for circadian light exposure calculation. However, in this case, theoretical activation of the ipRGCs by light (melanopic lux) was estimated from SPR data using the irradiance toolbox developed by CIE S 026/E:2018 (2018). Then, the melanopic yielded lux were correlated with recordings gathered by ACM circadian light sensor. Finally, Bland–Altman plots were constructed for both visible and circadian lights measured with SPR and ACM device, before and after infrared correction, to investigate any possible existence of bias among measurements.

### Statistical Analysis

Linear correlations and algorithm were calculated using R Core Team (2017). R: A language and environment for statistical computing (R Foundation for Statistical Computing, Vienna, Austria)^1^ Bland–Altman plots were constructed using Microsoft Excel 2010 software (Microsoft Corporation, Redmond, WA, United States).

## Results

The reliability of light measurements by the ACM light sensors for visible and circadian light was tested against SPR illuminance recordings both in lux and in logarithmic units for all lights here assessed. Correlation analysis between SPR and ACM illuminance measurements (in lux) for visible light (Table 1) resulted to be strong (*r* = 0.946) and highly significant (*p* < 0.0001) with a slope of 0.558. This correlation was even stronger when considering illuminance in log lux (slope = 0.867, *r* = 0.996, *p* < 0.0001).

**Table 1:** Correlation between spectroradiometer and Kronowise measurements for visible and circadian light according to CIE S 026 standard, *r* coefficient and *p*-value, and degrees of freedom (DF) for all light types together, for lights with low IR, for lights with high IR, for the Sun, and for recalculated visible and melanopic light.

Correlation			Formula	*r* coefficient	*p* value	DF
Spectroradiometer vs. Kronowise^®^	Visible light	All lights	*y* = 0.558*x*	0.946	<0.0001	35
		All lights (LOG)	*y* = 0.867*x*	0.996	<0.0001	35
		Low IR	*y* = 0.684*x*	0.997	<0.0001	27
		Low IR (LOG)	*y* = 0.893*x*	0.997	<0.0001	27
		High IR	*y* = 0.272*x*	0.999	<0.0001	3
		High IR (LOG)	*y* = 0.756*x*	0.992	<0.0005	3
		Sun	*y* = 0.297*x*	0.987	<0.005	3
		Sun (LOG)	*y* = 0.843*x*	0.999	<0.0001	3
		Calculated	*y* = 0.994*x*	0.997	<0.0001	35
		Calculated (LOG)	y = 1.025*x*	0.998	<0.0001	35
	Blue light	All lights	*y* = 1.324*x*	0.902	<0.0001	35
		All lights (LOG)	*y* = 1.029*x*	0.989	<0.0001	35
		Low IR	*y* = 1.883*x*	0.999	<0.0001	27
		Low IR (LOG)	*y* = 0.110*x*	0.995	<0.0001	27
		High IR	*y* = 0.310*x*	0.999	<0.0001	3
		High IR (LOG)	*y* = 0.753*x*	0.992	<0.001	3
		Sun	*y* = 0.878*x*	0.991	<0.001	3
		Sun (LOG)	*y* = 0.990*x*	0.999	<0.0001	3
		Calculated	*y* = 0.997*x*	0.998	<0.0001	35
		Calculated (LOG)	*y* = 1.020*x*	0.998	<0.0001	35

However, light sources with high infrared content do not behave according to the general equation and it has to be considered. For that, the IR/Full light ratio was calculated for all lights (Table 2) and three groups of light sources emerged: artificial lights with low IR content (IR/Full light < 0.1), artificial lights with high IR content (IR/Full light ≥ 0.6) and sun light (0.1 ≤ IR/Full light < 0.6).

**Table 2:** Infrared/Full light ratio (IR/Full) for lights tested at four different distances (D1 = 54 cm, D2 = 180 cm, D3 = 237 cm, and D4 = 480 cm).

	IR/Full					
Light	D1	D2	D3	D4	MEAN	SEM
RGB LED	0.013	0.009	0.012	0.011	0.011	0.001
RGV LED	0.015	0.013	0.013	0.013	0.013	0.001
5700 K LED	0.017	0.014	0.012	0.008	0.013	0.002
3000 K LED	0.026	0.021	0.019	0.019	0.021	0.002
Amber LED	0.027	0.03	0.032	0.032	0.03	0.001
Mercury vapor	0.025	0.024	0.026	0.024	0.025	0.001
Fluorescent	0.017	0.019	0.019	0.022	0.019	0.001
Sun	0.168	0.172	0.161	0.166	0.167	0.002
Incandescent	0.627	0.632	0.634	0.644	0.634	0.004

*Three groups of lights were obtained: artificial illuminants with low IR content (outlined by a red box), artificial illuminants with high IR content (green box) and the sunlight (purple box).*

When the IR/Full light ratio for visible light, in lux and in log lux, was considered and a specific linear equation per lighting groups was used, correlations between SPR and Kronowise^®^ for all lights improved considerably. It resulted to be stronger and with slope closer to 1 (slope = 0.994, *r* = 0.997, *p* < 0.0001 for lux units and slope = 1.025, *r* = 0.998, *p* < 0.0001 for log lux) than when the IR/Full light ratio was not considered (slope = 0.558, *r* = 0.946, *p* < 0.0001 for lux units and slope = 0.867, *r* = 0.996, *p* < 0.0001 for log lux) (Table 1).

The same process was applied to circadian light detected by the blue sensor. Correlation between SPR melanopic lux (CIE S 026/E:2018, 2018) and ACM with blue filter lux recordings resulted to be strong (slope = 1.324, *r* = 0.902 in lux and slope = 1.029, *r* = 0.989 in log lux) and significant (*p* < 0.0001 in both units). Again, the interference of infrared content slightly reduced the accuracy of the estimation. Therefore, correlations improved when the three lighting groups were considered (slope = 0.997, *r* = 0.998, *p* < 0.0001 for lux units and slope = 1.020, *r* = 0.998, *p* < 0.0001 for log lux).

About ACM device’s stability based on the incident angle, placing the light sensors under a diffusing surface allows the detection of light to exceed 80% of the maximum in a range of 50° of change in the incident light, and 50% of the maximum is detected in a range of 70° (Figure 5).

**FIGURE 7:**
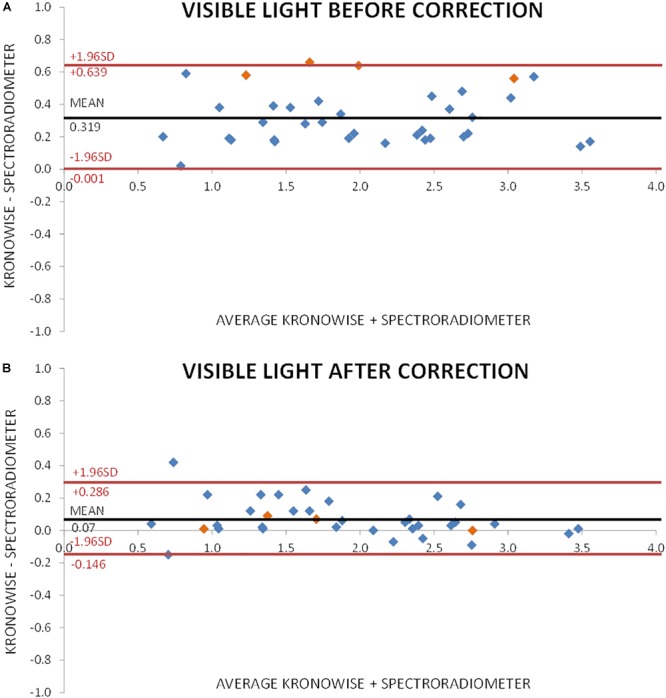
Bland–Altman plot comparing visible light intensity recorded by spectroradiometer and ACM. **(A)** Before correction for infrared content. Orange dots correspond to incandescent light and blue dots to low infrared content lights and sun light. **(B)** After correction for infrared content. Orange dots correspond to incandescent light and blue dots to low infrared content lights and sun light.

**FIGURE 8:**
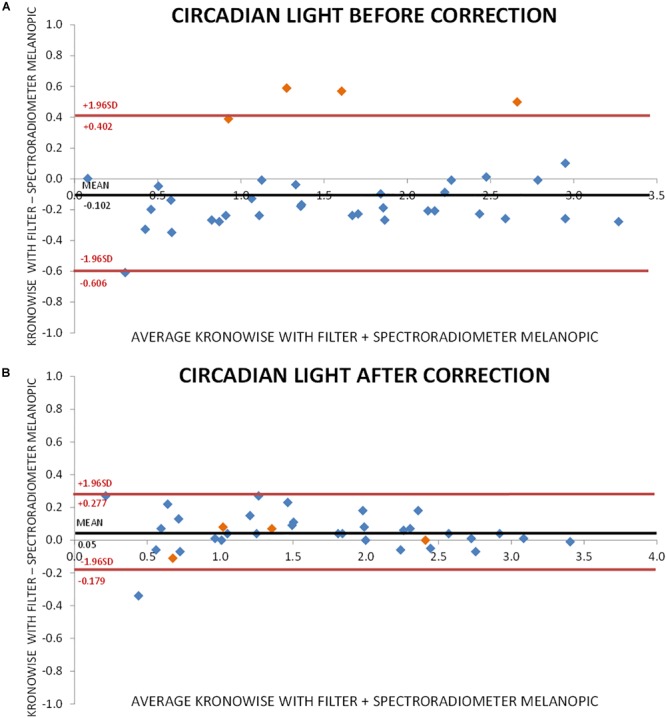
Bland–Altman plot comparing circadian light intensity according to CIE S 026 standard, recorded by spectroradiometer and ACM. **(A)** Before correction for infrared content. Orange dots correspond to incandescent light and blue dots to low infrared content lights and sun light. **(B)** After correction for infrared content. Orange dots correspond to incandescent light and blue dots to low infrared content lights and sun light.

Figure 7A shows the Bland–Altman plot for visible light measurements with SPR and ACM device before correction by infrared content. As it can be observed, illuminance data from Kronowise^®^ full spectrum sensor tended to overestimate light intensity when compared to SPR. This effect was even more pronounced for those light sources with high infrared content. Figure 7B represents the Bland–Altman plot for all illuminance visible measurements after correction by infrared content, and as it could be noticed, it improves considerably. The same occurs with the Bland–Altman plots for circadian light both before and after correction by infrared content (Figure 8A,B).

## Discussion

Here, we reported the spatial and spectral ability of a wrist worn ACM device provided with three light sensors that combines full spectrum, infrared and blue light simultaneous monitoring, allowing not only intensity and timing of visible and circadian light exposure to be evaluated, but also to infer light source and thus, differentiate between natural and artificial light exposure.

Due to the recent discovery of ipRGCs and their role in circadian photoreception (Brainard et al., 2001; Berson et al., 2002), it becomes necessary to develop practical and non-invasive tools to specifically differentiate its stimulation under free living conditions (Heil and Mathis, 2002; Sarabia et al., 2008), and not only consider light intensity when studying circadian system synchronization to light and its health consequences.

The integration in the same device of multiple sensors for detecting several circadian outputs as motor activity, body position, and wrist skin temperature rhythms together with individual exposure to visible and circadian light will facilitate understanding, diagnosis and treatment of those problems associated to circadian synchronization, increasingly frequent in developed societies.

In order to facilitate the usability of the ACM device under normal living conditions, it has been designed to be worn on the wrist, discarding other placements closer to the eyes but much more uncomfortable for the subjects. Although, at first instance, it could be argued that this position could affect the accuracy of lighting measurements, previous studies by Figueiro et al. (2013) have shown that differences in active circadian light exposure are surprisingly small, typically less than 10% on average, when data at eye and wrist level are compared. Thus, light exposure measurement in other places than the eye seems to be also a reliable method when assessing circadian light exposure.

To record visible light (400–700 nm), a combination of two sensors is used in Kronowise^®^, one of full spectrum ranging from 400 to 1100 nm and another for infrared light (from 700 to 1100 nm). The combination of these two measures allows, on one hand, to differentiate whether light source is natural or artificial and among these, if light is provided by sources with high or low content in infrared radiation, or by the sun, as those light spectra shown in Figure 6. The circadian-effective light is detected thanks to blue filter covering a second full light spectrum sensor. The selected blue filter shows a transmittance profile to sunlight that is highly coincident both with the spectral inhibition curve of melatonin (Brainard et al., 2001) and with the model of melanopsin sensitivity curve of the ipRGCs (Enezi et al., 2011), letting pass visible wavelengths between 380 and 590 nm and above 680 nm. Thus, the “dose” of active circadian light to which the subject is exposed to can be calculated without inferences from combination of blue and green detectors, as it happens with Actiwatch Spectrum (Cao et al., 2015). To date, and to our knowledge, only two ambulatory devices are available for the detection of visible and circadian light: the Daysimeter (Bierman et al., 2005) and Actiwatch Spectrum (Phillips). The Daysimeter, a head-mounted device, has been specifically designed for detecting light exposure, so its performance in this field is very interesting; however, the device only includes an accelerometer and must be placed close to the eyes, limiting seriously its ability to detect sleep and wakefulness states. In addition, and although its light sensor shows a sensitivity curve restricted to the photopic spectrum, it shows a significant drop between 550 and 600 nm, a band to which the human eye is sensitive (Dartnall et al., 1983; Roorda and Williams, 1999).

The second device, Actiwach Spectrum, was primarily designed for detecting sleep and wake rhythms (Young et al., 2009; Kripke et al., 2010), and later incorporated RGB detection. This latter consists of three color sensors for light in the long wavelength (∼600–700 nm), middle-wavelength (450–600 nm) and short-wavelength range (∼400–550 nm), which correspond to R, G, B spectral outputs and a broadband “white light” (W) output (Price et al., 2012; Cao et al., 2015). However, its sensitivity spectrum does not match the one for the human retina. It shows a bimodal pattern, with a main peak at short wavelengths and a secondary peak at long wavelengths. In contrast, in the 570–600 nm band, the device is practically non-sensitive. This band, apart from its sensitivity of human retina, is characteristic of discharge light sources such as fluorescent lamps (Figueiro et al., 2013). Consequently, the photometric measurements of these common light sources will be systematically biased with this device and circadian light stimulation must be indirectly deduced from data recorded by blue and green sensors.

This study possess a limitation since only one Kronowise^®^ ACM device and a single lighting source at a time was used, thus future studies should employ a combination of different artificial and natural lights to resemble natural conditions.

The ACM device here presented possesses a unique combination of sensors for simultaneous recordings of skin temperature, position, movement (circadian outputs), and exposure to visible light and circadian light (circadian inputs). Previous studies showed that the combination of skin temperature, actimetry and position provides a reliable evaluation of circadian system status, since it includes a variable with an endogenous component (skin temperature) but also variables reactive to behavioral demands (motor activity and body position) (Ortiz-Tudela et al., 2010, 2014; Bonmati-Carrion et al., 2014b). Considering that the most powerful *zeitgeber* for circadian entrainment is the light-dark cycle (Roenneberg and Foster, 1997; Roenneberg et al., 2003), its simultaneous recording with other output signal seems a must to obtain an integrative assessment on the circadian function. In this sense, Kronowise^®^ can constitute a useful and comfortable tool to deep our knowledge on light synchronization effects while people maintain their usual lifestyle.

## Conclusion

Our results have proved the reliability and sensitivity of the ACM device Kronowise^®^ to assess exposure to light of different spectra. Besides, our algorithm seems valid to calculate, directly from Kronowise^®^ data, visible and circadian light exposure, and discriminating among different light types, providing a more detailed light exposure individual history.

The use of calibrated ACM devices as the one presented here, combining circadian rhythms and exposure to visible and circadian light simultaneous monitoring, will contribute to the advance on understanding of light effects on the circadian system synchronization and their association with human health. Its non-invasive and ease of use nature will facilitate its employment for monitoring large populations while maintaining their normal lifestyle.

## Data Availability

The datasets generated for this study are available on request to the corresponding author.

## Author Contributions

RA-P and MB-C acquired the data. RA-P, MB-C, and JM analyzed the data. All authors conceived and designed the study, wrote the first draft of the manuscript, contributed to manuscript revision, and read and approved the submitted version.

## Conflict of Interest Statement

MR and JM are founding partners of Kronohealth SL, a spin-off company, also participated and co-founded by the University of Murcia. Kronohealth has not contributed to finance this study. The remaining authors declare that the research was conducted in the absence of any commercial or financial relationships that could be construed as a potential conflict of interest.

## Footnotes

1 https://www.R-project.org/

## References

[B1] Berger A. M. Wielgus K. Hertzog M. Fischer P. Farr L. (2010). Patterns of circadian activity rhythms and their relationships with fatigue and anxiety / depression in women treated with breast cancer adjuvant chemotherapy. *Support. Care Cancer* 18 105–114. 10.1007/s00520-009-0636-0 19381692

[B2] Berson D. M. Dunn F. A. Takao M. (2002). Phototransduction by retinal ganglion cells that set the circadian clock. *Science* 295 1070–1073. 10.1126/science.1067262 11834835

[B3] Bierman A. Klein T. R. Rea M. S. (2005). The daysimeter: a device for measuring optical radiation as a stimulus for the human circadian system. *Meas. Sci. Technol.* 16 2292–2299. 10.1088/0957-0233/16/11/023

[B4] Bonmati-Carrion M. A. Arguelles-Prieto R. Martinez-Madrid M. J. Reiter R. Hardeland R. Rol M. A. (2014a). Protecting the melatonin rhythm through circadian healthy light exposure. *Int. J. Mol. Sci.* 15 23448–23500. 10.3390/ijms151223448 25526564 PMC4284776

[B5] Bonmati-Carrion M. A. Middleton B. Revell V. Skene D. J. Rol M. A. Madrid J. A. (2014b). Circadian phase asessment by ambulatory monitoring in humans: correlation with dim light melatonin onset. *Chronobiol. Int.* 31 37–51. 10.3109/07420528.2013.820740 24164100

[B6] Brainard G. C. Hanifin J. P. Greeson J. M. Byrne B. Glickman G. Gerner E. (2001). Action spectrum for melatonin regulation in humans: evidence for a novel circadian photoreceptor. *J. Neurosci.* 21 6405–6412. 10.1523/JNEUROSCI.21-16-06405.2001 11487664 PMC6763155

[B7] Cao D. Barrionuevo P. A. Sciences V. (2015). Estimating photoreceptor excitations from spectral outputs of a personal light exposure measurement device. *Chronobiol. Int.* 32 270–280. 10.3109/07420528.2014.966269.Estimating 25290040 PMC4355054

[B8] CIE (1951). *Commission Internationale de l’Eclairage Proceedings.* Cambridge: Cambridge University Press.

[B9] CIE S 026/E:2018 (2018). *CIE System for Metrology of Optical Radiation for ipRGC-Influenced Responses to Light.* Vienna: CIE.

[B10] Dartnall H. J. Bowmaker J. K. Mollon J. D. (1983). Human visual pigments: microspectrophotometric results from the eyes of seven persons. *Proc. R. Soc. London. Ser. B. Biol. Sci.* 220 115–130. 6140680 10.1098/rspb.1983.0091

[B11] Enezi J. Al Revell V. Brown T. Wynne J. Schlangen L. Lucas R. (2011). A “melanopic” spectral efficiency function predicts the sensitivity of melanopsin photoreceptors to polychromatic lights. *J. Biol. Rhythms* 26 314–323. 10.1177/0748730411409719 21775290

[B12] Erren T. C. Reiter R. J. (2009). Light Hygiene: time to make preventive use of insights - old and new - into the nexus of the drug light, melatonin, clocks, chronodisruption and public health. *Med. Hypotheses* 73 537–541. 10.1016/j.mehy.2009.06.003 19586725

[B13] Figueiro M. G. Hamner R. Bierman A. Rea M. S. (2013). Comparisons of three practical field devices used to measure personal light exposures and activity levels. *Light. Res. Technol.* 45 421–434. 10.1177/1477153512450453 24443644 PMC3892948

[B14] Garaulet M. Madrid J. A. (2010). Chronobiological aspects of nutrition, metabolic syndrome and obesity. *Adv. Drug Deliv. Rev.* 62 967–978. 10.1016/j.addr.2010.05.005 20580916

[B15] Heil D. P. Mathis S. R. (2002). Characterizing free-living light exposure using a wrist-worn light monitor. *Appl. Ergon.* 33 357–363. 10.1016/S0003-6870(02)00007-8 12160339

[B16] Kolodyazhniy V. Späti J. Frey S. Götz T. Wirz-justice A. Kräuchi K. (2011). Estimation of human circadian phase via a multi-channel ambulatory monitoring system and a multiple regression model. *J. Biol. Rhythms* 26 55–67. 10.1177/0748730410391619 21252366

[B17] Kripke D. F. Hahn E. K. Grizas A. P. Wadiak K. E. P. H. Loving R. T. Poceta J. S. (2010). Wrist actigraphic scoring for sleep laboratory patients: algorithm development. *J. Sleep Res.* 19 612–619. 10.1111/j.1365-2869.2010.00835.x 20408923

[B18] Lemmer B. Scholtze J. Schmitt J. (2016). Circadian rhythms in blood pressure, heart rate, hormones, and on polysomnographic parameters in severe obstructive sleep apnea syndrome patients: effect of continuous positive airway pressure. *Blood Press. Monit.* 21 136–143. 10.1097/MBP.0000000000000173 26683380

[B19] Madrid-Navarro C. J. Escamilla-Sevilla F. Mínguez-Castellanos A. Campos M. Ruiz-Abellán F. Madrid J. A. (2018). Multidimensional circadian monitoring by wearable biosensors in Parkinson’s disease. *Front. Neurol.* 9:157. 10.3389/fneur.2018.00157 29632508 PMC5879441

[B20] Navara K. J. Nelson R. J. (2007). The dark side of light at night: physiological, epidemiological, and ecological consequences. *J. Pineal Res.* 43 215–224. 10.1111/j.1600-079X.2007.00473.x 17803517

[B21] Ortiz-Tudela E. Bonmati-Carrion M. L. De la Fuente M. Mendiola P. (2012). La cronodisrupción como causa de envejecimiento. *Rev. Esp. Geriatr. Gerontol.* 47 168–173. 10.1016/j.regg.2011.09.013 22177973

[B22] Ortiz-Tudela E. Martinez-Nicolas A. Albares J. Segarra F. Campos M. Estivill E. (2014). Ambulatory circadian monitoring (ACM) based on thermometry, motor activity and body position (TAP): a comparison with polysomnography. *Physiol. Behav.* 126 30–38. 10.1016/j.physbeh.2013.12.009 24398067

[B23] Ortiz-Tudela E. Martinez-Nicolas A. Campos M. Rol M. Á Madrid J. A. (2010). A new integrated variable based on thermometry, actimetry and body position (TAP) to evaluate circadian system status in humans. *PLoS Comput. Biol.* 6:e1000996. 10.1371/journal.pcbi.1000996 21085644 PMC2978699

[B24] Panda S. Hogenesch J. B. Kay S. A. (2002). Circadian rhythms from flies to human. *Nature* 417 329–335. 10.1038/417329a 12015613

[B25] Pauley S. M. (2004). Lighting for the human circadian clock: recent research indicates that lighting has become a public health issue. *Med. Hypotheses* 63 588–596. 10.1016/j.mehy.2004.03.020 15325001

[B26] Price L. L. A. Khazova M. Hagan J. B. O. (2012). Performance assessment of commercial circadian personal exposure devices. *Light. Res. Technol.* 44 17–26. 10.1177/1477153511433171

[B27] Reiter R. J. Tan D.-X. Korkmaz A. Erren T. C. Piekarski C. Tamura H. (2012). Light at night, chronodisruption, melatonin suppression, and cancer risk: a review. *Crit. Rev. Oncog.* 13 303–328. 10.1615/critrevoncog.v13.i4.30 18540832

[B28] Roenneberg T. Daan S. Merrow M. (2003). The art of entrainment. *J. Biol. Rhythms* 18 183–194. 12828276 10.1177/0748730403018003001

[B29] Roenneberg T. Foster R. G. (1997). Twilight times: light and the circadian system. *Photochem. Photobiol.* 66 549–561. 9383985 10.1111/j.1751-1097.1997.tb03188.x

[B30] Roorda A. Williams D. R. (1999). The arrangement of the three cone classes in the living human eye. *Nature* 397 520–522. 10028967 10.1038/17383

[B31] Sarabia J. A. Rol M. A. Mendiola P. Madrid J. A. (2008). Circadian rhythm of wrist temperature in normal-living subjects A candidate of new index of the circadian system. *Physiol. Behav.* 95 570–580. 10.1016/j.physbeh.2008.08.005 18761026

[B32] Vos J. J. (1978). Colorimetric and photometric properties of a 2° fundamental observer. *Color Res. Appl.* 3 125–128. 10.1002/col.5080030309

[B33] Young K. Kripke D. F. Poceta J. S. Shadan F. Jamil S. M. Cronin J. W. (2009). Evaluation of immobility time for sleep latency in actigraphy. *Sleep Med.* 10 621–625. 10.1016/j.sleep.2008.07.009 19103508

